# Reduced uremic metabolites are prominent feature of sarcopenia, distinct from antioxidative markers for frailty

**DOI:** 10.18632/aging.203498

**Published:** 2021-09-07

**Authors:** Masahiro Kameda, Takayuki Teruya, Mitsuhiro Yanagida, Hiroshi Kondoh

**Affiliations:** 1 Geriatric Unit, Graduate School of Medicine, Kyoto University, Kyoto, Japan; 2 G0 Cell Unit, Okinawa Institute of Science and Technology Graduate University (OIST), Okinawa, Japan

**Keywords:** sarcopenia, muscle mass, metabolomics, frailty, uremic metabolites

## Abstract

Due to global aging, frailty and sarcopenia are increasing. Sarcopenia is defined as loss of volume and strength of skeletal muscle in elderlies, while frailty involves multiple domains of aging-related dysfunction, impaired cognition, hypomobility, and decreased social activity. However, little is known about the metabolic basis of sarcopenia, either shared with or discrete from frailty. Here we analyzed comprehensive metabolomic data of human blood in relation to sarcopenia, previously collected from 19 elderly participants in our frailty study. Among 131 metabolites, we identified 22 sarcopenia markers, distinct from 15 frailty markers, mainly including antioxidants, although sarcopenia overlaps clinically with physical frailty. Notably, 21 metabolites that decline in sarcopenia or low SMI are uremic compounds that increase in kidney dysfunction. These comprise TCA cycle, urea cycle, nitrogen, and methylated metabolites. Sarcopenia markers imply a close link between muscle and kidney function, while frailty markers define a state vulnerable to oxidative stress.

## INTRODUCTION

Due to the wave of global aging, aging-related diseases among the elderly are increasing: hypertension, diabetes, atherosclerosis, osteoporosis, dementia, cancer, etc. Frailty and sarcopenia are also well known as aging-related diseases [1, 2]. Both diseases are increasing with estimated global populations of about 120 million and 90 million individuals, respectively [3, 4]. The clinical evaluation for sarcopenia is different from that for frailty. Sarcopenia is defined as a loss of skeletal muscle and muscle strength in the elderly [5], while frailty is a state of vulnerability to several stressors, due to declined function or impairment of organs and tissues during aging [1]. Frailty encompasses multiple domains of aging, including cognitive impairment, hypomobility, and decreased social activity [1, 6].

Frail patients overlap with 20–70% of sarcopenic populations [7–10]. In addition, both sarcopenia and frailty significantly affect the general status of the elderly, including mortality, hospitalization rate, falling, and necessity of long-term care [5, 6, 11]. This is partly because sarcopenic patients share clinical features with physical frailty, a subtype of frailty [1, 5]. Three major tools are applied for the diagnosis of frailty: 1) The physical frailty model known as the Fried Cardiovascular Health Study Index (CHS) [1], 2) the deficit accumulation model, covering multimorbidity, known as the Rockwood Frailty Index [12], and 3) the Edmonton Frailty Scale (EFS) or Tilburg Frailty Indicator, a mixed physical and psychosocial model [13, 14]. Thus, the Rockwood Frailty Index and EFS are distinct from physical frailty in evaluating cognitive or social function. Several reports suggest the involvement of cytokines in sarcopenia [15–17]. However, little is known about the metabolic basis which may be shared or discrete in sarcopenia and frailty.

Metabolomics is a newly developed branch of chemistry that detects and quantifies small molecules, called metabolites, using methods such as liquid chromatography- mass spectrometry (LC-MS) [18]. Metabolites are generated in cells and tissues through their metabolic activities. They include amino acids, carbohydrates and organic acids, nitrogen compounds, purines and pyrimidines, lipids, antioxidants, etc. Metabolomics reveal complex, highly integrated biological processes. Human blood reflects physiological and pathophysiological states influenced by heredity, epigenetics, and disease, as well as by physiological or homeostatic responses, lifestyle, and nutrition [18–20]. Blood metabolomics have been utilized to reveal pathology and to identify diagnostic biomarkers [21–23].

Recently, we established reproducible and quantitative analyses for metabolomics using whole blood [24]. Although many studies have examined human blood serum or plasma, our whole blood analysis covers metabolites from both cellular and non-cellular compartments [24]. This approach has been validated in several comprehensive, non-targeted studies for comparisons between yeast and human blood [25], aging metabolites [26], and fasting compounds [27]. In addition, our whole blood metabolomics have identified 15 frailty markers, including 10 antioxidants, based on EFS diagnostic tools [28]. Interestingly, although the average ages of both frail and non-frail populations were more than 80 years in our study, 5 of 15 frailty-related metabolites overlapped with aging markers [26] [28], indicating an intriguing metabolic link between frailty and human aging.

Here previous metabolomic data from the study of frailty diagnosed using the EFS are analyzed, based on sarcopenic diagnoses in the same group [28]. In sharp contrast to the 15 frailty markers, including antioxidants, we identified 22 sarcopenia markers, comprising TCA cycle compounds, urea cycle compounds, muscle or nitrogen metabolites, and methylated metabolites. Interestingly, most metabolites that decreased in sarcopenia or low SMI are uremic compounds that increase as a result of kidney impairment. Thus, metabolite profiles in sarcopenia are largely distinct from those of frailty.

## RESULTS

**Figure 1:**
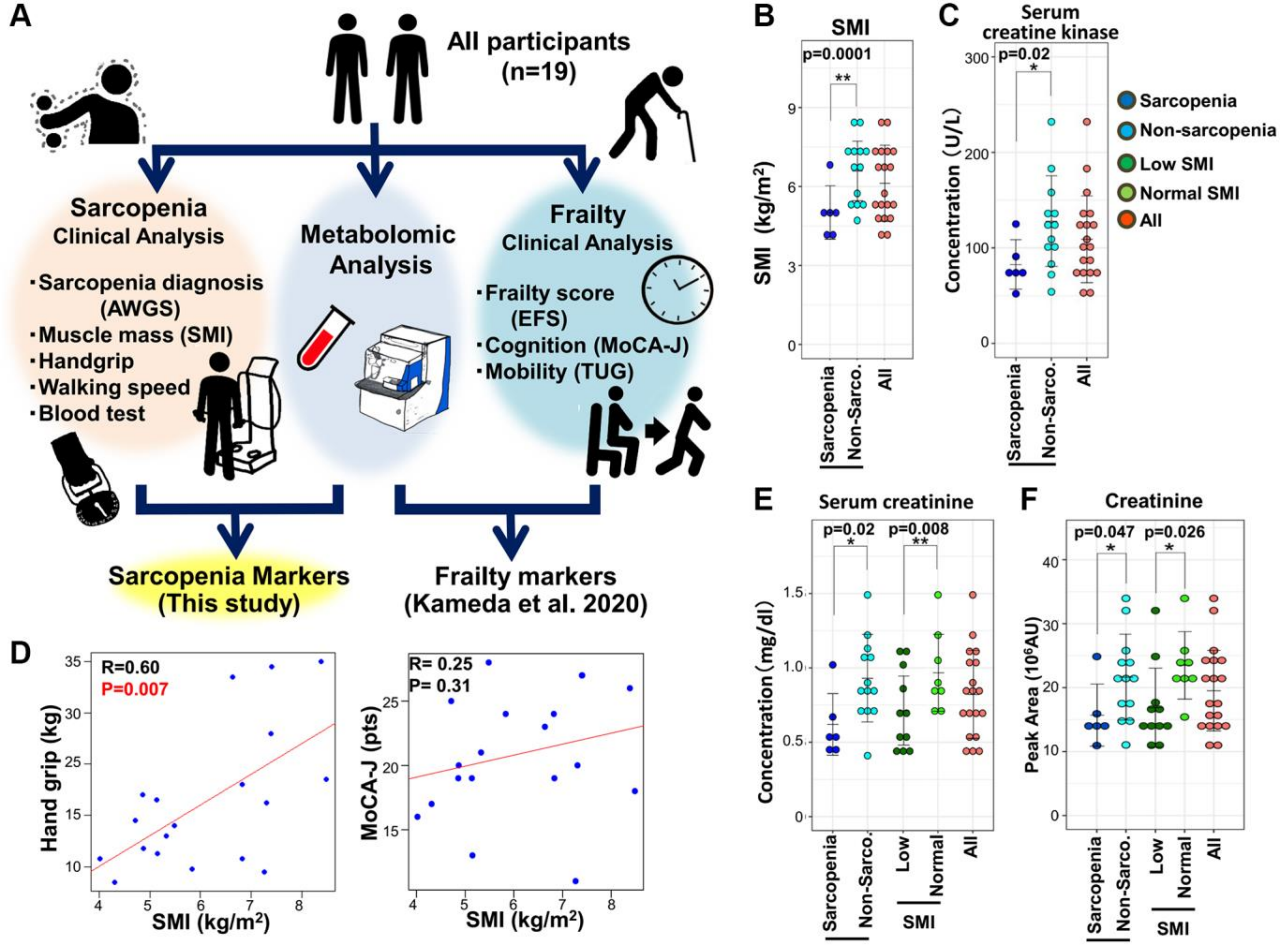
**The metabolomic study of sarcopenia.** (**A**) Overview of the study protocol. All participants were clinically assessed, and their blood was evaluated by untargeted whole-blood metabolomics. This study was conducted using previously reported clinical data from 19 elderly participants who were also assessed for sarcopenia. (**B**) Comparison of SMI between sarcopenic and non-sarcopenic subjects. SMI was significantly decreased in the sarcopenia group. (**C**) Pearson's correlation of the linear model between hand grip and SMI (left panel). The correlation coefficient between hand grip and SMI was statistically significant (R = 0.60, *p* = 0.007). The correlation between MoCA-J and SMI was not statistically significant (R = 0.25, *p* = 0.31). (**D**) Serum creatine kinase levels decreased significantly in sarcopenia. (**E**) Serum creatinine decreased significantly in sarcopenia and the low-SMI group. (**F**) Metabolomic analysis by LC-MS detected a significant decrease of creatinine in sarcopenia and the low-SMI group. ^*^p < 0.05. Error bars represent means ± SD. ^**^p < 0.01. Error bars represent means ± SD.

We previously reported findings of non-targeted comprehensive metabolomic analysis of whole blood based on frailty diagnosis using the EFS in 19 elderly participants (7 males and 12 females; average age; 84.2 ± 6.9 years) [28] (Figure 1A). As this study also included sarcopenic profiles in the same population, here we analyzed metabolite profiles from the same data, based on individual diagnoses of sarcopenia (Figure 1A). Clinical features for frailty and results of blood tests were shown in Supplementary Table 1 in Kameda et al. PNAS 2020. Sarcopenia was diagnosed based on metrics of the Asian Working Group for Sarcopenia (AWGS), which includes hand grip strength, a 10-meter speed-walking test, and skeletal muscle index (SMI) among patients over 65 years old [5]. SMI was recorded by bioelectric impedance analysis [29]. Among individuals with decreased SMI, patients with decreased hand grip strength or walking speed were diagnosed as sarcopenic.

Clinical attributes of all participants for sarcopenia are summarized in Supplementary Table 1. Among 19 participants, 6 individuals (average age; 85.0 ± 8.6) were diagnosed as sarcopenic, while 13 (average age; 83.8 ± 6.3) were not (Supplementary Table 1). Non-sarcopenia group included 5 cases of pre-sarcopenia, 5 cases of dynapenia, and 5 cases of frailty. Sarcopenia group included 4 cases of frailty. SMI and BMI were significantly decreased in sarcopenia, while hand grip strength was not (Figure 1B, Supplementary Figure 1, and Supplementary Table 1). Handgrip strength was significantly correlated with SMI (Figure 1C). Calf circumference was significantly decreased in sarcopenia and low SMI group. Other clinical parameters were comparable between the two groups. Among results of blood tests, both serum creatinine and creatine kinase were significantly decreased in sarcopenia (Figure 1D, Figure 1E, and Supplementary Table 1), consistent with previous findings. However, serum creatinine and creatine kinase in frailty were comparable to those in non-frailty [28]. Thus, regarding clinical markers such as creatinine and creatine kinase (CK), sarcopenia is distinct from other muscle degenerative diseases accompanied by increases in these markers [30] and also distinct from frailty. Lower CK levels in sarcopenia would be caused by the decreased turnover of muscle tissues or by decreased physical activity in patients. Regarding SMI, 11 individuals displayed low SMI (average score; 5.2 ± 0.9), while 8 were normal (average 7.4 ± 0.8). BMI, serum creatinine, and BUN were significantly decreased in the low-SMI group (Figure 1E, and Supplementary Table 1). However, we noticed that cognitive function in sarcopenic patients is comparable to that in subjects without sarcopenia, assessed using the Japanese version of the Montreal Cognitive Assessment, (MoCA-J) (Figure 1C, and Supplementary Figure 1). Thus, in sharp contrast to the close correlation between frailty and impaired cognition, sarcopenia appears unlinked to cognitive impairment [7].

All metabolomic profiles in these participants were previously analyzed by LC-MS [28]. The 131 metabolites detected were confirmed using standard compounds or by MS/MS, as previously reported [28]. We performed a comprehensive assessment of 131 metabolites based on the diagnosis of sarcopenia (Supplementary Data 1). We identified 22 compounds as sarcopenic markers (Supplementary Table 2), which did not overlap with 15 frailty markers in the same dataset [28]. Among these 22 compounds, 21 metabolites (acetyl-carnitine, dimethyl-proline, phenylalanine, dimethyl-arginine, N1-methyl-histidine, isovaleryl-carnitine, myo-inositol, creatinine, pantothenate, hypoxanthine, dimethyl-guanosine, N1-methyl-adenosine, 2-oxoglutarate, pentose-phosphate, succinate, N-acetyl-glutamate, quinolinic acid, 4-guanidinobutanoate, N1-methyl-guanosine, trimethyl-tyrosine, and cis-aconitate) decrease significantly in sarcopenia, while aspartate increases. In addition, comparisons between low- and normal-SMI groups identified 10 SMI markers: urate, butyro-betaine, dimethyl-arginine, N1-methyl-histidine, isovaleryl-carnitine, creatinine, hippurate, dimethyl-guanosine, 2-oxoglutarate, and cis-aconitate (Supplementary Table 3). All 10 SMI markers are significantly decreased in the low-SMI group. Thirteen of 22 sarcopenia markers and all 10 SMI markers are significantly correlated with SMI (Supplementary Table 4). As three SMI markers (urate, butyro-betaine, and hippurate) did not overlap with sarcopenia markers, a total of 25 metabolites are identified as sarcopenia-related markers.

**Figure 2:**
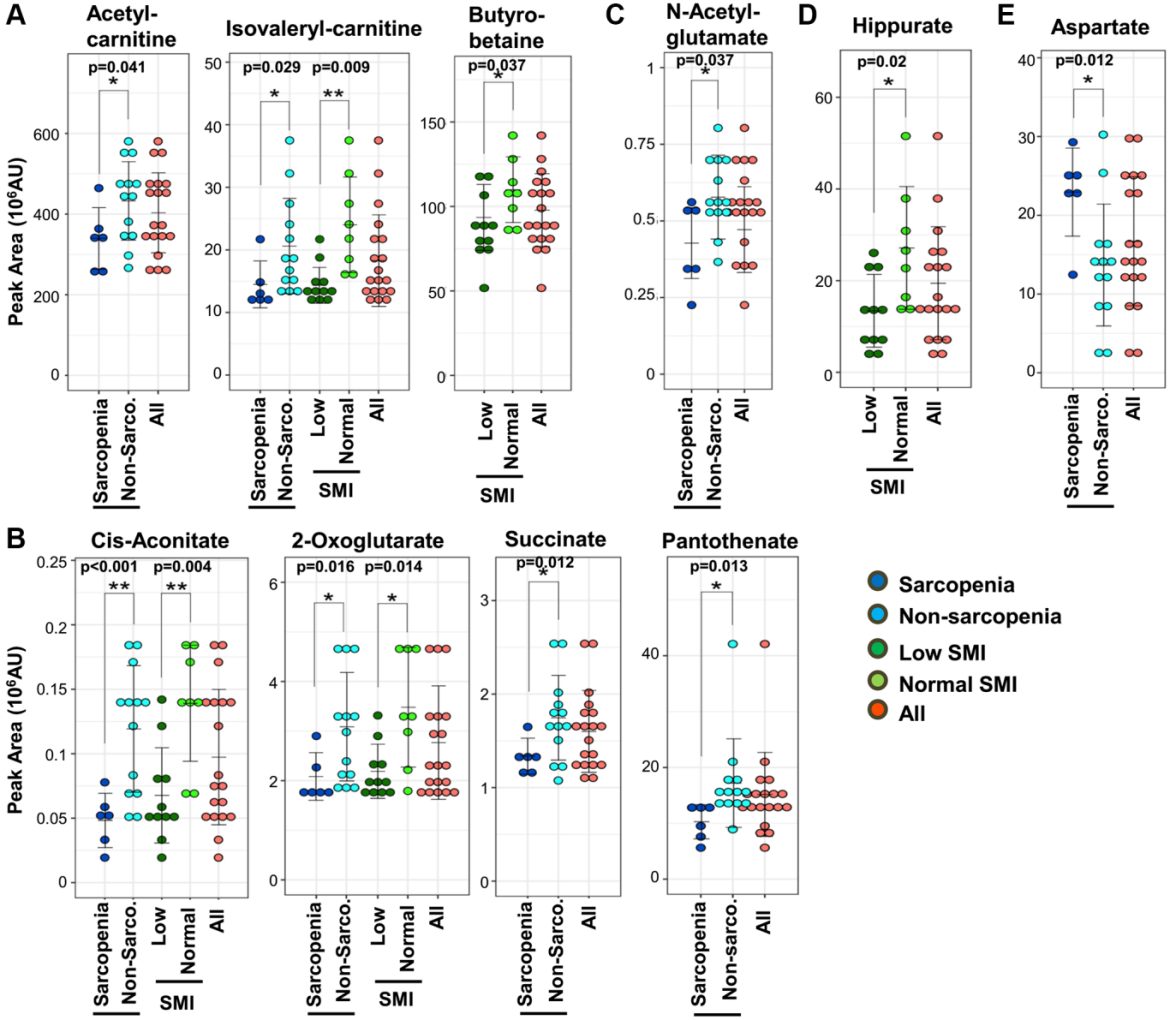
**Ten mitochondrial metabolites are diagnostic for sarcopenia.** (**A**) Three short-chain carnitines and their derivatives (acetyl-carnitine, isovaleryl-carnitine, and butyro-betaine) decreased significantly in sarcopenia. Isovaleryl-carnitine was significantly decreased in the low-SMI group. (**B**) Four TCA-related metabolites (2-oxoglutarate, cis-aconitate, succinate, and pantothenate) decreased significantly in sarcopenia. 2-oxoglutarate and cis-aconitate were significantly reduced in the low-SMI group. (**C**) N-acetyl-glutamate, which is related to the urea cycle, was significantly diminished in sarcopenia. (**D**) Hippurate, which is related to ammonia synthesis, was significantly decreased in the low-SMI group. (**E**) Aspartate, which is involved in the mitochondrial malate-aspartate shuttle, was significantly increased in sarcopenia. ^*^p < 0.05 Error bars represent means ± SD. ^**^p < 0.01. Error bars represent means ± SD.

First, consistent with findings in blood tests (Figure 1E), metabolomic analysis detected a decline of creatinine both in sarcopenia and in the low-SMI group (Figure 1F). In clinical practice, creatinine is well known as a marker of both kidney disease and muscle mass [31]. We observed that 10 sarcopenia-related markers pertain to mitochondria: 8 sarcopenia markers (acetyl-carnitine, isovaleryl-carnitine, 2-oxoglutarate, cis-aconitate, succinate, aspartate, N-acetyl-glutamate, and pantothenate) and 5 SMI markers (isovaleryl-carnitine, butyro-betaine, 2-oxoglutarate, cis-aconitate, and hippurate) (Figure 2). Except aspartate, the other 9 mitochondrial metabolites are significantly decreased. Short-chain carnitines (acetyl- and isovaleryl-carnitine) supply acetyl-CoA to mitochondria (Figure 2A). Butyro-betaine is a precursor of carnitine. TCA metabolites (cis-aconitate, 2-oxoglutarate, and succinate) are involved in energy production in the mitochondrial matrix (Figure 2B). Pantothenate is the precursor of CoA. Mitochondria are also involved in the urea cycle, one of the substrates of which is N-acetyl-glutamate (Figure 2C) [32]. Hippurate is generated in mitochondria during ammonia synthesis (Figure 2D) [33]. In the previous metabolomic study of frailty, the assessment using the timed-up-and-go (TUG) test also identified hippurate and isovaleryl-carnitine as hypomobility markers [28]. Some mitochondrial metabolites displayed high correlation coefficients: 2-oxoglutarate and cis-aconitate (R = 0.66, *p* = 0.002), 2-oxoglutarate and succinate (R = 0.75, *p* = 0.0002), cis-aconitate and succinate (R = 0.59, *p* = 0.007). Aspartate was significantly increased in sarcopenia (Figure 2E) and negatively correlated with mitochondrial metabolites (2-oxoglutarate; R = −0.56, *p* = 0.012, and succinate; R = −0.60, *p* = 0.0002) (Supplementary Figure 2). Aspartate is utilized in the mitochondrial malate-aspartate shuttle.

**Figure 3:**
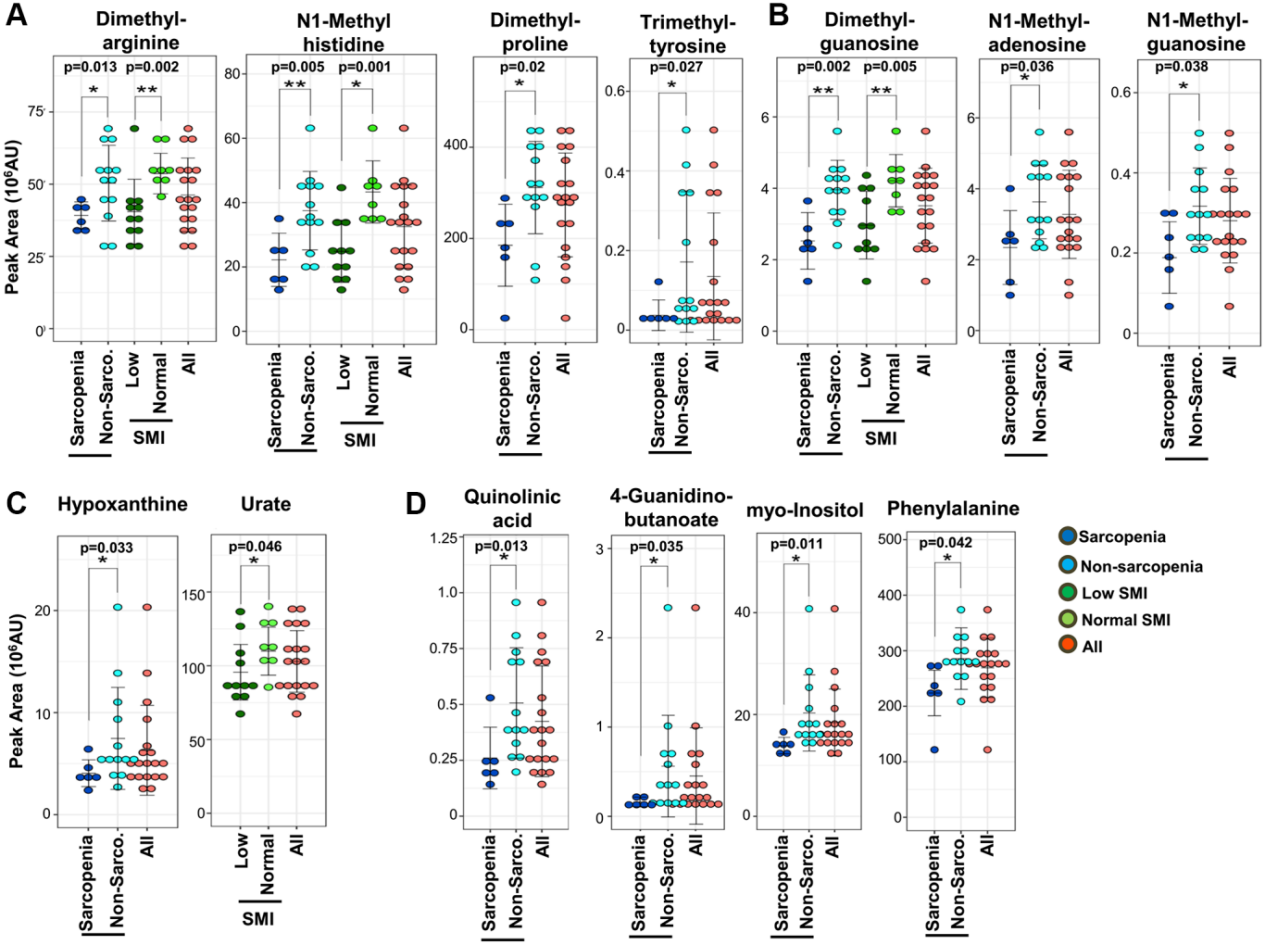
**Sarcopenic markers related to methylated metabolites and kidney disease.** (**A**) Five methylated amino acids and their derivatives (dimethyl-arginine, N1-methyl-histidine, dimethyl-proline, trimethyl-tyrosine, and butyro-betaine) were significantly decreased in sarcopenia or the low-SMI group. 4 metabolites (N1-methyl-histidine, trimethyl-tyrosine, dimethyl-arginine, and dimethyl-proline) were significantly decreased in the sarcopenia group. Three metabolites (N1-methyl-histidine, dimethyl-arginine, butyro-betaine) were significantly decreased in the low-SMI group. (**B**) Three methylated nucleotides (dimethyl-guanosine, N1-methyl-adenosine, and N1-methyl-guanosine) were significantly decreased in sarcopenia. Dimethyl-guanosine was significantly decreased in the low-SMI group. (**C**) Two uremic markers related to purine metabolism (hypoxanthine and urate) were involved in sarcopenia. Hypoxanthine was significantly decreased in sarcopenia, whereas urate was significantly decreased in the low-SMI group. (**D**) Eight metabolites related to kidney disease (creatinine, dimethyl-arginine, dimethyl-guanosine, N1-methyl-guanosine, quinolinic acid, 4-guanidinobutanoate, myo-inositol, and phenylalanine) were significantly decreased in sarcopenia. Three metabolites (creatinine, dimethyl-arginine, and dimethyl-guanosine) were also significantly decreased in the low-SMI group. ^*^p < 0.05 Error bars represent means ± SD. ^**^p < 0.01. Error bars represent means ± SD.

Second, it is noteworthy that 8 methylated metabolites decrease in sarcopenia (Figure 3A and 3B): 5 methylated compounds related to amino acids (N1-methyl-histidine, trimethyl-tyrosine, dimethyl-arginine, dimethyl-proline, and butyro-betaine) (Figure 3A) and 3 methylated nucleotides (N1-methyl-adenosine, N1-methyl-guanosine, and dimethyl-guanosine) (Figure 3B). Additionally, pentose phosphate metabolites, related to synthesis of nucleotides, decrease in sarcopenia (Supplementary Figure 3A).

Third, we observed that 10 decreased metabolites overlapped with previously reported markers for uremia or kidney disease: creatinine, dimethyl-arginine, dimethyl-guanosine, quinolinic acid, N1-methyl-guanosine, hypoxanthine, urate, 4-guanidinobutanoate, myo-inositol, and phenylalanine (Figure 3C and 3D) [31, 34–38]. Creatinine is well known as a marker both for kidney disease and muscle mass in clinical practice [31]. Hypoxanthine is the precursor of urate, which was also reported as a frailty marker [28] (Figure 3C). ATP levels in muscle reportedly decline during aging [39], while in our whole blood metabolomics we observed that blood ATP levels were less affected in sarcopenia. The decrease of urate and hypoxanthine in blood in sarcopenia may reflect reduced ATP metabolism in aging muscles. 4-Guanidinobutanoate is related to NH_3_ metabolism (Figure 3D). Moreover, other decreased metabolites related to mitochondria (cis-aconitate, 2-oxoglutarate, succinate, pantothenate, N-acetyl-glutamate, acetyl-carnitine, isovaleryl-carnitine, butyro-betaine, and hippurate) and methylation (N1-methyl-adenosine and N1-methyl-histidine) are also increased in patients with kidney disease. Thus, in total, 21 metabolites that decreased in sarcopenia or low SMI are uremic compounds. These uremic compounds are statistically correlated. Creatinine is significantly correlated with other uremia markers; hypoxanthine (R = 0.76, *p* = 0.0001), urate (R = 0.71, *p* = 0.0007), dimethyl-arginine (R = 0.73, *p* = 0.0003), dimethyl-guanosine (R = 0.56, *p* = 0.0136), N1-methyl-guanosine (R = 0.67, *p* = 0.002), quinolinic acid (R = 0.71, *p* = 0.0006), and myo-inositol (R = 0.75, *p* = 0.0002) (Supplementary Figure 2). Moreover, these kidney markers are significantly correlated with other sarcopenic markers (isovaleryl-carnitine, butyro-betaine, cis-aconitate, succinate, N-acetyl-glutamate, and N1-methyl-histidine) (Supplementary Figure 2). We observed the gender differences in 7 metabolites, which are significantly decreased in female; 23.89 ± 7.85, 15.47 ± 5.00, *p* = 0.03, in isovaleryl-carnitine, 24.79 ± 3.59, 16.61 ± 6.15, *p* = 0.002 in creatinine, 54.51 ± 10.42, 42.07 ± 11.02, *p* = 0.03 in dimethyl-arginine, 4.07 ± 0.43, 3.15 ± 1.14, *p* = 0.02 in dimethyl-guanosine, 27.06 ± 13.54, 13.86 ± 8.87, *p* = 0.05 in hippurate, 0.35 ± 0.10, 0.24 ± 0.09, *p* = 0.04 in N1-methyl-guanosine, and 119.58 ± 19.81, 93.90 ± 16.89, *p* = 0.02 in urate (mean ± SD of peak area in male and female, and *p*-value of *t*-test, respectively).

**Figure 4:**
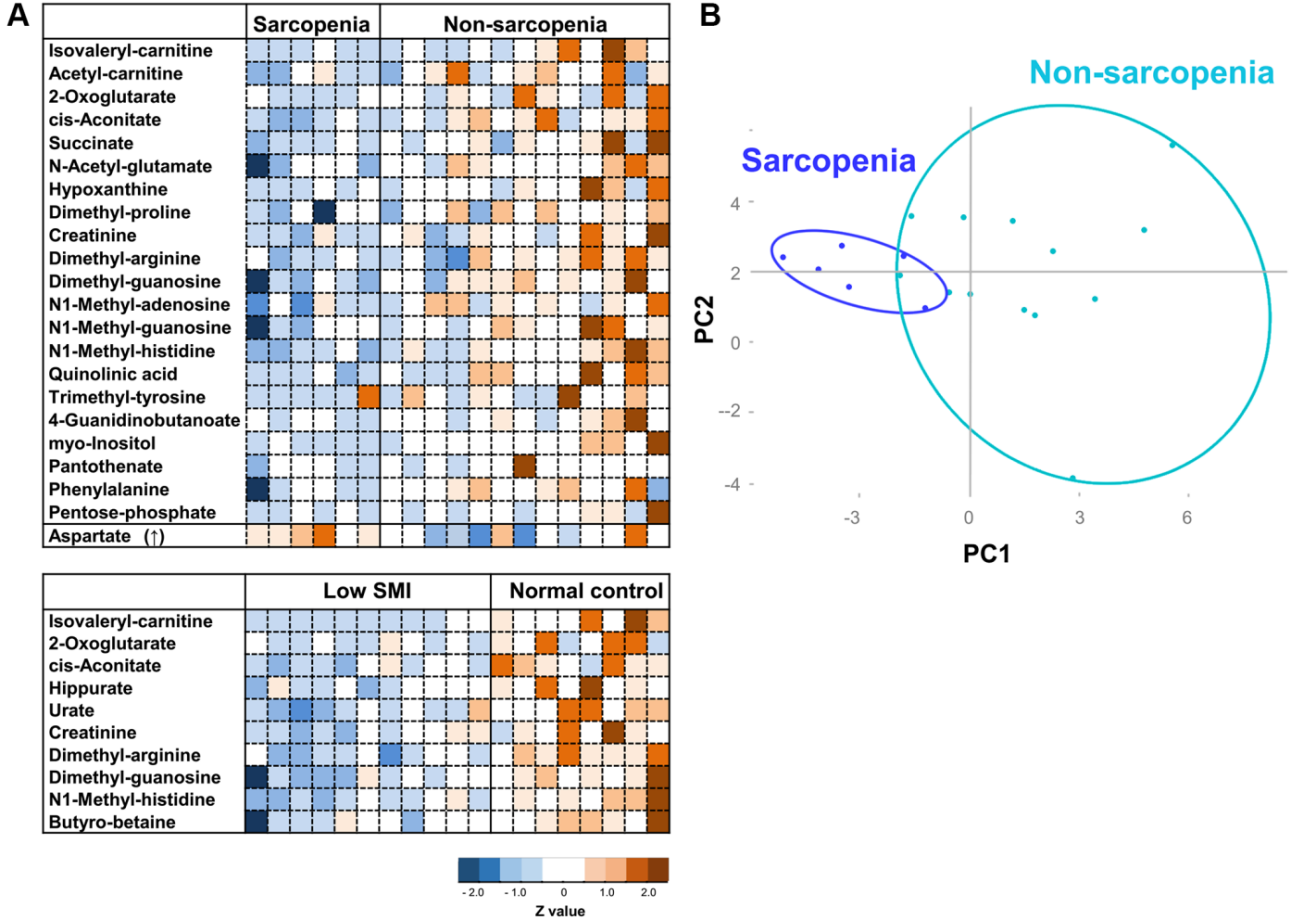
**Heatmap analysis and PCA for sarcopenia.** (**A**) Heatmap analysis of metabolites involved in sarcopenia (top panel), and SMI (bottom). The heat map shows Z-scores of peak areas from LC-MS analysis. (**B**) PCA plot of 19 elderly participants. 22 sarcopenia markers were analyzed.

Finally, we addressed the question of whether these sarcopenia-related metabolites are useful for detection of sarcopenia. As we noticed that 22 sarcopenia markers are distinct from 15 previously reported frailty markers (Figure 4) [28], we assessed the correlation analysis between 25 sarcopenia-related metabolites and Edmonton frail scale (EFS), and that between 22 frailty-related markers and SMI. Three sarcopenia-related markers (isovaleryl-carnitine, hippurate, and urate) were significantly correlated with EFS (Supplementary Table 4), while eight frailty-related markers (acetyl-carnosine, urate, 1,5-anhydroglucitol, proline, methionine, leucine, isovaleryl-carnitine, hippurate) were significantly correlated with SMI (Supplementary Table 5). These results suggest that sarcopenia-related or frailty metabolites partly correlate with the diagnostic parameters for frailty (EFS) or sarcopenia (SMI), respectively. However, heatmap analysis and principal component analysis (PCA) using sarcopenia-related metabolites indicated much closer interrelation between sarcopenia and sarcopenia-related metabolites. Heatmap comparisons of 22 sarcopenia markers revealed their distinct distributions in sarcopenic and non-sarcopenic persons (Figure 4A upper panel). Similar results were observed regarding 10 SMI markers among low-SMI and control groups (Figure 4A lower panel). Next, we applied PCA, based on 22 sarcopenia markers. PCA distinguished sarcopenia patients from non-sarcopenia controls (Figure 4B). However, PCA with sarcopenia markers did not distinguish the frail population from non-frail controls (Supplementary Figure 3B).

## DISCUSSION

**Figure 5:**
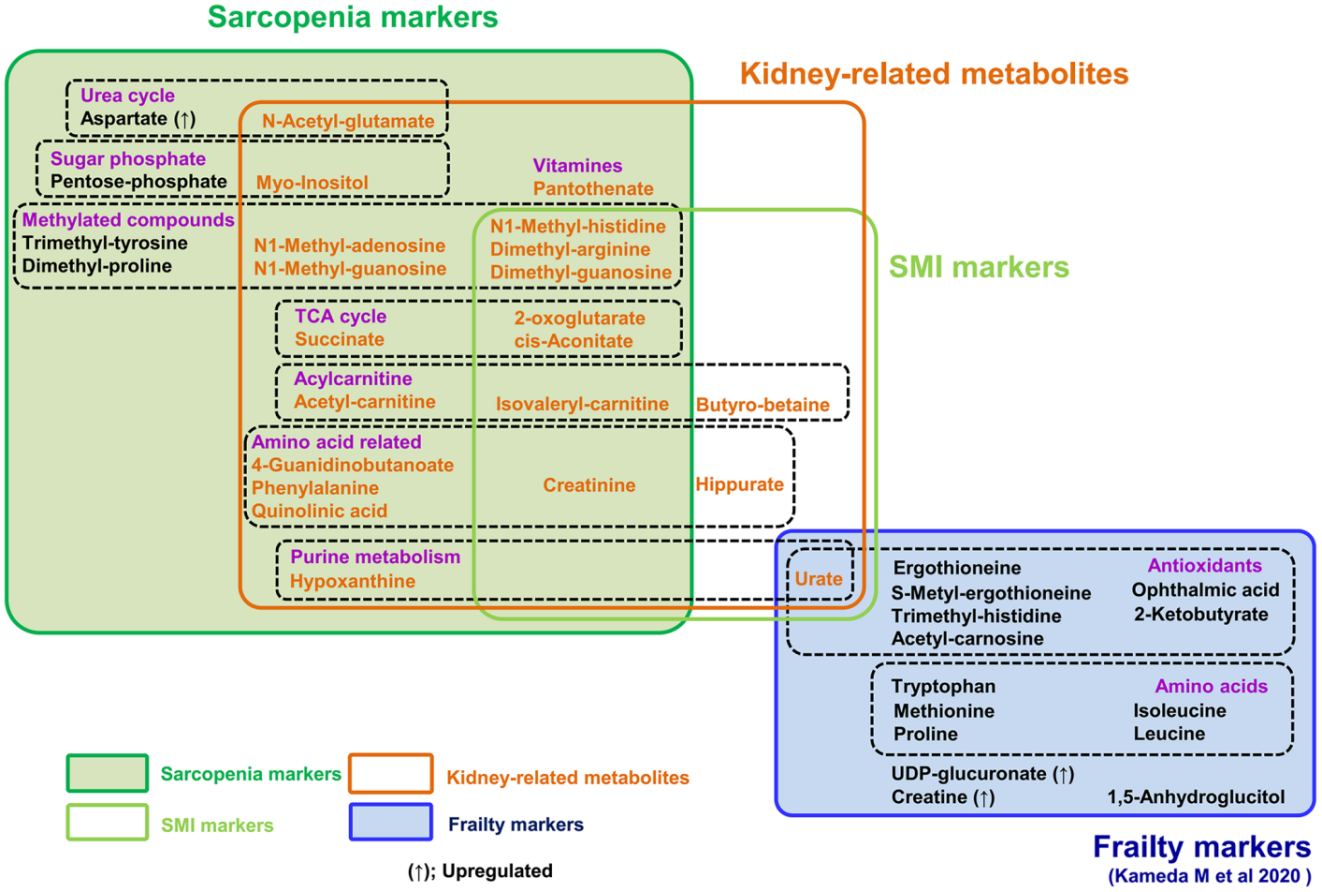
**Summary of 25 metabolites related to sarcopenia and 15 frailty markers.** 15 frailty markers (blue box) and 22 sarcopenia markers (dark green box) are presented. There is no overlap among them. 10 metabolites are muscle mass-related markers (light green box). Seven of the 15 frailty markers were antioxidants; however, sarcopenia markers include no antioxidants. Seven metabolites (isovaleryl-carnitine, 2-oxoglutarate, cis-aconitate, creatinine, dimethyl-arginine, dimethyl-guanosine, and N1-methyl-histidine) are both sarcopenia and muscle mass-related markers. 21 metabolites in orange are kidney-related markers.

Here non-targeted comprehensive metabolomic analysis of whole blood identified 25 sarcopenia-related metabolites: 22 sarcopenia markers and 10 SMI markers that overlap. These 25 markers include metabolites related to mitochondria, kidney function, nitrogen metabolism, and methylated compounds. It is noteworthy that 22 sarcopenia markers are distinct from 15 previously reported frailty markers (summarized in Figure 5), although clinical and metabolomic information were extracted from the same datasets. Thus, our metabolomic analysis revealed previously unknown aspects of the metabolite profile of sarcopenia. These metabolites could be developed into the future clinical use, as some metabolites, such as leucine and isoleucine, were effective to improve muscle quality [40].

It is well known that frailty and sarcopenia share several clinical features, although their definition and diagnostic criteria are distinct. Frailty displays complex domains, physical and social impairment, and diminished cognition, due to vulnerability to stressors in aged organs and tissues. Sarcopenia is defined as muscle aging, displaying decreased muscle mass and strength. Thus, both disease states are deeply affected by organismal aging, but the underlying metabolic bases are still unclear. Our metabolomic study evaluated both frailty and sarcopenia in the same participants. In this setting, we observed that no metabolites overlapped between frailty and sarcopenia (Figure 5) [28], although the markers for sarcopenia and frailty partly correlate with the diagnostic index for each others. Indeed, while 10 metabolites for antioxidation were identified as frailty markers diagnosed using the EFS [28], no antioxidant was included among 22 sarcopenic metabolites. Moreover, PCA of sarcopenia metabolites clearly distinguished sarcopenia from non-sarcopenia, although not frailty from non-frailty. Thus, their metabolite profiles partly overlap with each other, the diagnostic evaluation by those metabolites are much different. While our findings overlap partly with several other works [41–44], the difference in metabolite markers was noted. Such gaps are probably due to the difference in nutritional status (e.g., high BMI) [41], average age, and study design including whole blood metabolome. Although several metabolomics studies successfully reported findings with small group [45], it is important to verify these markers with larger subjects, e.g., by targeted metabolomics, in the near future.

Strikingly, we observed that compounds that decrease in sarcopenia largely overlap with those that increase in kidney diseases. In addition to the list of 10 sarcopenia markers relevant to kidney function (Figure 3), several reports suggest that other sarcopenic metabolites, relevant to mitochondria (9) and methylation (6), are also increased in uremia or kidney disease [31, 34, 37, 46–48]. Thus, the majority of decreased markers in sarcopenia or low SMI (21 of 24 metabolites) overlap with uremic compounds, which increase in renal dysfunction or uremia (Figure 5). It was also well known that creatinine, a kidney biomarker, declines during muscle loss [31]. Our findings suggest the possibility that waste actively generated via muscle metabolism could be a major burden for kidneys, failure of which results in increased levels of these sarcopenic markers. Alternatively, blood sarcopenia markers might decline by their enhanced excretion from kidney in sarcopenia.

Although other muscle diseases, e.g., muscle dystrophy, are frequently accompanied by increased markers for muscle degeneration, we observed that the majority of sarcopenic metabolites decrease, in addition to creatinine. Mitochondria-related metabolites, including short-chain carnitines, TCA metabolites, and urea cycle metabolites, decrease significantly in sarcopenia. Mitochondrial dysfunction in muscles during sarcopenia is well established in experimental models [49–51]. Consistently, recent comparative RNA analysis in muscle biopsy also identified mitochondrial dysfunction in sarcopenia [52]. Since RBCs in blood do not contain mitochondria, the decline in mitochondrial compounds reflects mitochondrial activity in muscle. Moreover, the decrease of several methylated compounds is an unexpected feature of sarcopenia. S-adenosyl methionine (SAM) is the major methyl-group donor in methylation of DNA, histones, proteins, lipids, and RNA [53]. However, as SAM does not decrease significantly in sarcopenia, some other muscle- or tissue-specific methylation pathway component may be impaired in sarcopenia. TRMT10C and TRMT5 mediate tRNA methylation by generating N1-methyladenosine and N1-methylguanosine, respectively. Interestingly, enzymatic mutations of TRMT10C and TRMT5 causes mitochondrial respiratory chain defects [54–56]. N1-methyladenosine, N1-methylguanosine, and dimethyl-guanosine, known as indices of RNA methylation [57], decrease in sarcopenia. These methylated blood metabolites may have a pathological link to mitochondrial or muscle dysfunction.

Notably, these 22 sarcopenia markers are largely distinct from 15 frailty markers in the same patients, suggesting that metabolic profiles distinguish sarcopenia from frailty. Thus, sarcopenia can be characterized as muscle aging with a decrease of metabolites for mitochondria, muscle, kidney, and methylation, in sharp contrast to the decrease of metabolites for antioxidation in frailty [28]. These findings help not only our understanding of pathogenesis of sarcopenia and frailty, but also future development of clinical applications.

## MATERIALS AND METHODS

### Clinical assessment

All clinical data were recorded at Kyoto University Hospital. Medical interviews, physical examinations, and blood tests were executed for nineteen elderly participants. Patients who were bedridden, or who had kidney failure (elevation of serum creatinine, over 2.0 mg/dL), or liver disease (increased serum GOT and GPT, over 50 U/L), were excluded from this study. Diagnosis of sarcopenia was performed using AWGS 2014 [5], which consists of muscle mass evaluation, a 10-m speed-walking evaluation, and a hand grip strength test. SMI was measured by Inbody 720 (South Korea). SMI below 7.0 kg/m^2^ in males and 5.7 kg/m^2^ in females was classified as decreased SMI. Patients who had an apparent risk of falling in the 10-m walking test or whose walking speed was below 0.8 m/sec, were considered hypomobile. Participants were asked to walk at a comfortable speed on a 12-m straight walkway, including 1 m for acceleration and deceleration. Stopwatch was used for the start and end points to record the time taken to walk 10 m, and habitual gait speed was measured by calculating this in meters per second [58]. Hand grip strength below 26 kg in male and 18 kg in female was considered decreased muscle strength. Handgrip was measured using a Smedley-type hand-held dynamometer (Matsumiya Ika Seiki Seisakusho Co., Ltd., Tokyo, Japan) [59]. Calf circumference was assessed, one of the clinical parameters in AWGS 2019 [59]. The total number of iADL impairment in EFS was scored.

### Blood sample preparation for metabolomic analysis

Blood samples were prepared for metabolomic analysis as previously reported [25, 26]. Blood for clinical tests and metabolomic analysis was collected at the laboratory of Kyoto University Hospital in the morning. All participants were asked not to have breakfast to ensure overnight fasting for at least 12 hours until the time of blood sampling. Participants were encouraged to spend their time normally and to drink beverages without calories. Since some metabolites are labile, blood samples were rapidly quenched at −40°C in methanol to guarantee quick sample processing [26]. Ten nmol of 4-(2-hydroxyethyl)-1-piperazineethanesulfonic acid (HEPES) and piperazine-N, N’-bis (2-ethanesulfonic acid) (PIPES) were added to each sample, to serve as internal standards.

### LC-MS conditions

Untargeted, comprehensive analysis by LC-MS was executed as previously reported [25, 26]. LC-MS data were acquired using an Ultimate 3000 DGP-3600RS liquid chromatograph (Thermo Fisher Scientific, Waltham, MA, USA) combined with an LTQ Orbitrap mass spectrometer (Thermo Fisher Scientific, Waltham, MA, USA). LC separation utilized a ZIC-pHILIC column (Merck SeQuant, Umeå, Sweden, 150 mm × 2.1 mm, 5 μm particle size). The mobile phase consisted of ammonium carbonate buffer (10 mM, pH 9.3) and acetonitrile. Gradient elution from 80 to 20% acetonitrile over 30 min at a flow rate of 100 μL/min was performed. An electrospray ionization (ESI) source was utilized for MS detection. An injection of 1 μL was carried out twice for each sample, once with the ESI in positive ionization mode and once in negative mode. Ion spray was set to 4.0 and 2.8 kV (positive or negative ESI, respectively), while the temperature of the capillary was kept to 300 or 350°C. Nitrogen gas was utilized as a carrier of ionized metabolites. We ran the mass spectrometer in full scanning mode with a 100–1000 m/z range and with MS/MS fragmentation scanning in an automatic data-dependent manner.

### LC-MS data processing and analysis

MZmine 2 (version 2.29) software (mzmine.github.io) was utilized to measure peak areas for metabolites [60]. Isotopic peaks were removed. Lists of peaks for individual samples were arranged, according to their retention times and corresponding m/z. 131 metabolites were identified for each sample by comparing retention times and m/z values of peaks with those of standards (Supplementary Data 1) [25, 26]. If no standard compound was accessible, metabolites were identified by analyzing MS/MS spectra (MS/MS). Then, all data acquired were converted into a spreadsheet, followed by evaluation with R statistical software (http://www.r-project.org). Student’s *T* test was performed to evaluate statistical significance of differences between groups (significance was set at *p* < 0.05) and its 95% confidence interval. The ordinary least squares method was used to assess linear regression. Pearson's correlation was performed to evaluate correlations between metabolites and clinical data (assuming *p* < 0.05). Principal component analysis was performed to visualize the metabolomic model.

### Data availability

Raw LC-MS data in mzML format are accessible from the MetaboLights repository (http://www.ebi.ac.uk/metabolights). The study identifier is MTBLS3341.

### Ethics statement

All participants signed written informed consent forms prior to examination, in accordance with the Declaration of Helsinki. Experiments were performed in agreement with relevant rules and official guidelines in Japan. The study protocol was approved both by the Human Research Ethics Committee of Kyoto University and by the Review Committee on Human Subjects Research at OIST.

## Supplementary Materials

Supplementary Figures

Supplementary Tables

Supplementary Data 1
