# Supplementary material for: Reduced uremic metabolites are prominent feature of sarcopenia, distinct from antioxidative markers for frailty
Source: Aging (Albany NY). 2021 Sep 7;13(17):20915–34. doi: 10.18632/aging.203498 (PMC8457568; doi:10.18632/aging.203498)
Supplement: Supplementary Figures [file aging-13-203498-s001.pdf]

SUPPLEMENTARY FIGURES

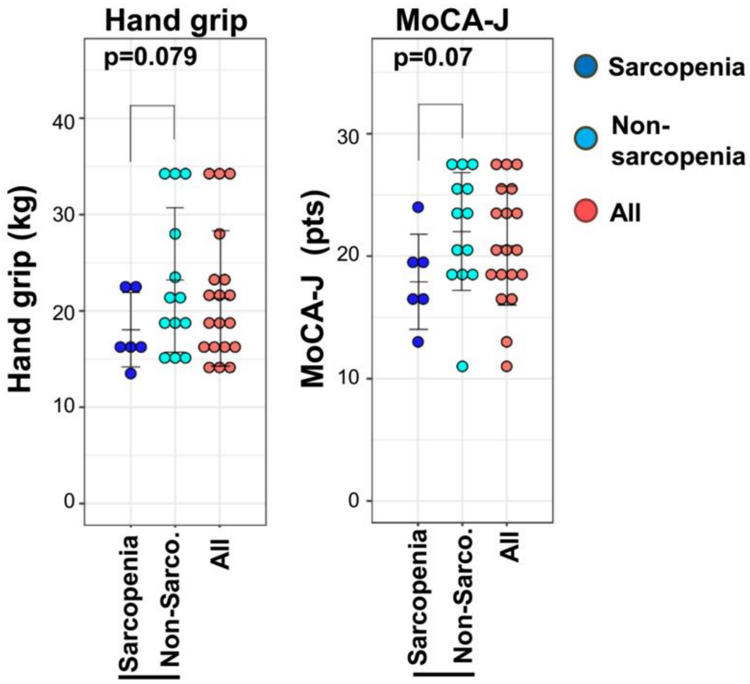

Supplementary Figure 1. Supplemental clinical data between sarcopenia and non-sarcopenia. Comparison of handgrip strength (left panel) and MoCA-J (right panel) between sarcopenia and non-sarcopenia were shown.

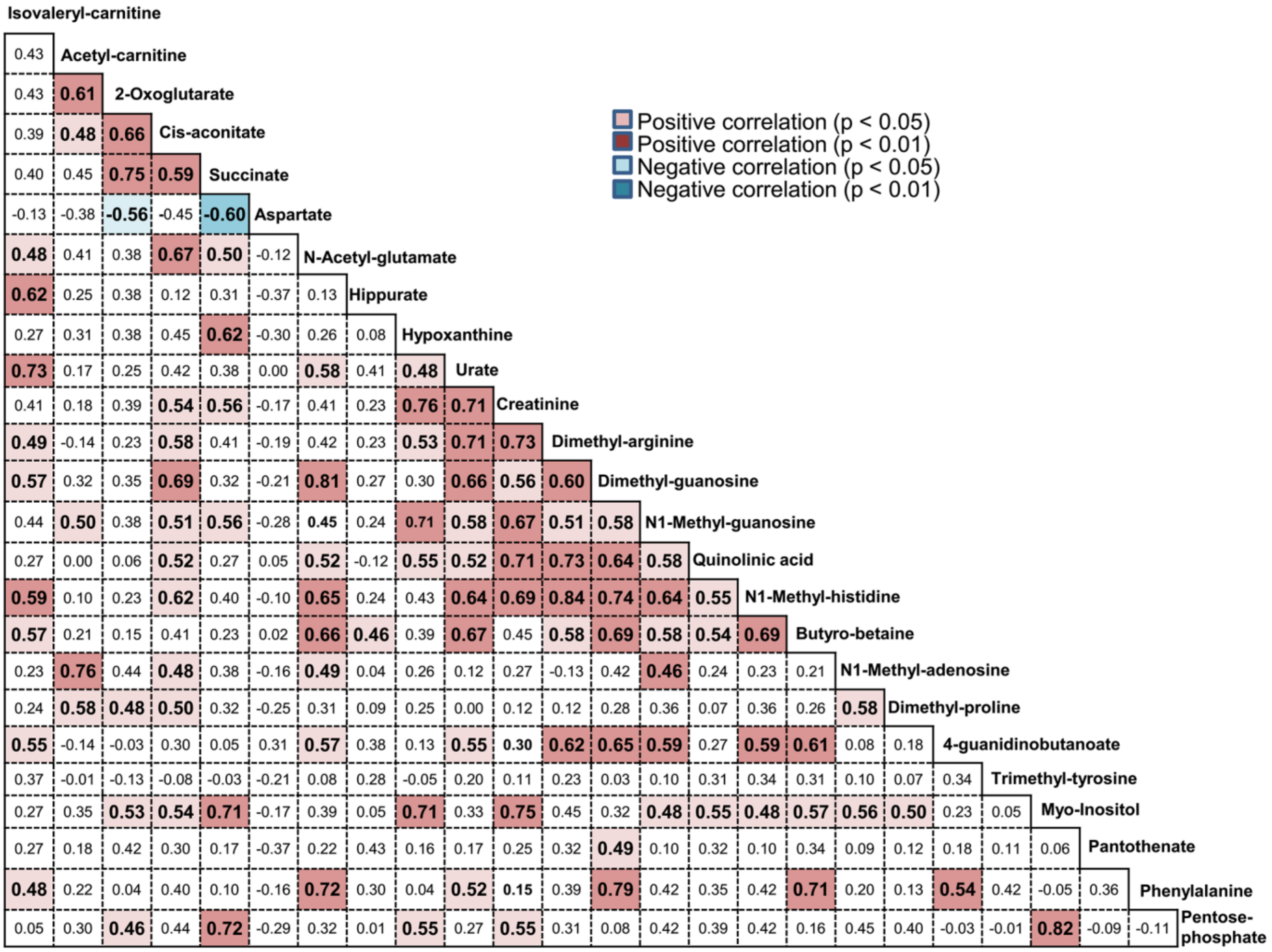

Supplementary Figure 2. Blood metabolites significantly affected in sarcopenia and muscle mass. Pearson's correlation analysis for 25 metabolites, relevant to sarcopenia or SMI. Positive and negative correlations are shown in brown and blue, respectively. Significant correlations between TCA cycle metabolites (cis-aconitate, 2-oxoglutarate, and succinate) or kidney markers (creatinine, dimethyl-arginine, dimethyl-guanosine, urate, hypoxanthine, N1-methyl-guanosine, and quinolinic acid) were observed.

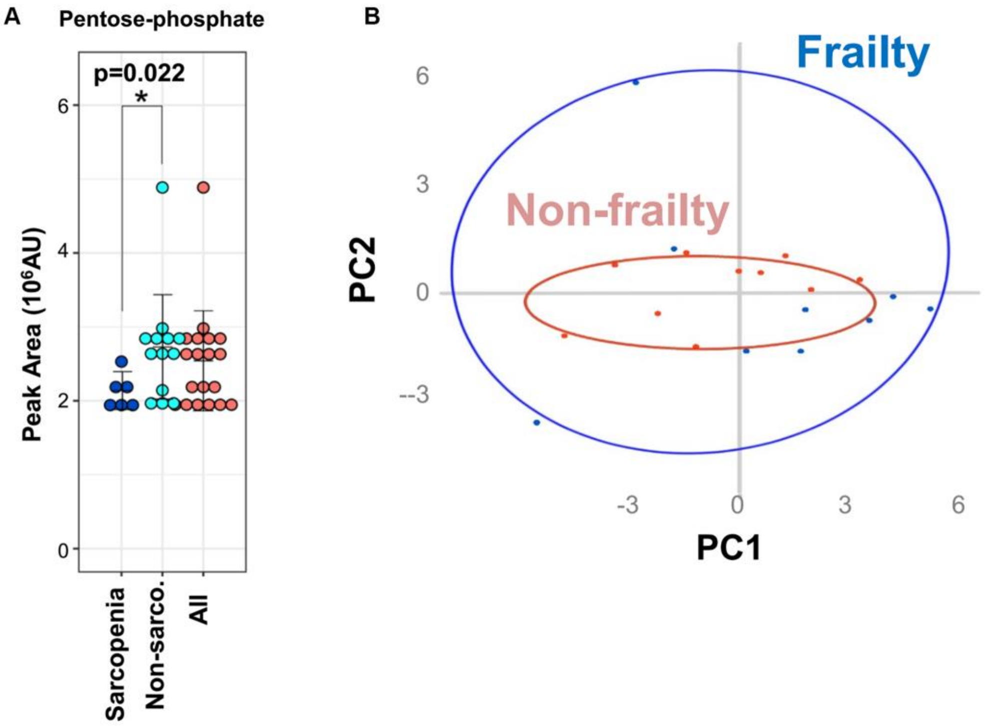

Supplementary Figure 3. Pentose-phosphates as sarcopenic markers and PCA for frailty using sarcopenia markers. (**A**) Pentose-phosphates were significantly decreased in sarcopenia. Error bars represent means ± SD. Asterisks indicate significant difference ($^{*}p < 0.05$). (**B**) PCA with 22 sarcopenia markers did not distinguish frailty patients from non-frailty controls.
