# Supplementary material for: Reduced uremic metabolites are prominent feature of sarcopenia, distinct from antioxidative markers for frailty
Source: Aging (Albany NY). 2021 Sep 7;13(17):20915–34. doi: 10.18632/aging.203498 (PMC8457568; doi:10.18632/aging.203498)
Supplement: Supplementary Tables [file aging-13-203498-s002.pdf]

SUPPLEMENTARY TABLES

Supplementary Table 1. Clinical parameters of human subjects.

	Sarcopenia (*N* = 6)	Non-Sarcopenia (*N* = 13)	95% CI of the difference	*P*-value	Reduced SMI (*N* = 11)	Control (*N* = 8)	95% CI of the difference	*p*-value
Age	85.0 ± 8.6	83.8 ± 6.3	–7.92 to 10.38	0.76	85.0 ± 8.6	83.8 ± 6.3	–7.92 to 10.38	0.76
Gender (Male; Female)	1;5	6;7			2;9	5;3		
SMI (kg/m^2)	5.0 ± 1.0	6.6 ± 1.2	–2.77 to –0.48	*0.01	5.2 ± 0.9	7.4 ± 0.8	–2.99 to –1.31	**0.0005
Handgrip(kg)	18.0 ± 4.0	23.0 ± 7.4	–10.53 to 0.66	0.08	21.1 ± 5.4	23.4 ± 8.4	–10.89 to 4.0	0.51
TUG(sec.)	23.5 ± 13.3	12.2 ± 6.5	–2.68 to 25.26	0.1	18.1 ± 11.4	12.6 ± 8.3	–4.10 to 14.95	0.27
Walking Speed (m/sec.)	1.07 ± 0.33	1.21 ± 0.40	–0.61 to 0.33	0.53	1.09 ± 0.26	1.27 ± 0.48	–0.57 to 0.21	0.37
BMI(kg/m^2)	20.0 ± 2.3	23.3 ± 3.6	–6.15 to –0.33	*0.03	20.7 ± 2.2	24.4 ± 4.0	–7.20 to –0.26	*0.04
Fat mass (kg)	12.7 ± 2.9	15.4 ± 5.7	–7.98 to 2.59	0.19	13.4 ± 2.7	16.0 ± 7.2	–7.60 to 2.33	0.35
Calf circumference(cm)	29.5 ± 4.1	33.5 ± 3.2	–7.65 to –0.42	*0.03	30.1 ± 3.1	35.3 ± 2.5	–8.12 to –2.42	**0.001
MoCA-J	18.2 ± 3.8	22.2 ± 4.8	–8.40 to 0.42	0.07	20.5 ± 4.3	21.5 ± 5.6	–6.17 to 4.08	0.66
Systolic BP(mmHg)	140.5 ± 10.4	136.5 ± 18.2	–9.95 to 18.03	0.55	143.9 ± 13.7	129.3 ± 14.2	0.40 to 28.92	*0.04
Diastolic BP(mmHg)	77.7 ± 6.8	77.3 ± 14.4	–9.89 to 10.61	0.94	77.2 ± 13.7	77.8 ± 10.1	–12.49 to 11.36	0.92
iADL	3.7 ± 3.1	2.0 ± 2.3	–1.05 to 4.38	0.28	2.7 ± 2.7	2.3 ± 2.7	–2.19 to 3.14	0.71
CRE□ mg/dl□	0.61 ± 0.22	0.92 ± 0.28	–0.57 to 0.06	*0.02	0.68 ± 0.23	1.03 ± 0.25	–0.59 to -0.11	**0.008
BUN□ mg/dl□	15.8 ± 4.8	19.7 ± 6.4	–9.67 to 1.75	0.16	15.6 ± 3.8	22.4 ± 6.6	–12.59 to –1.03	*0.025
CK□ U/L□	81.7 ± 24.6	124.4 ± 49.7	–77.50 to –7.33	*0.03	103.1 ± 54.2	121.3 ± 33.4	–59.69 to 23.62	0.39
AST（U/L□	20.8 ± 2.5	22.8 ± 4.8	–5.46 to 1.59	0.26	22.9 ± 4.7	21.1 ± 3.6	–2.20 to 5.77	0.36
ALT（U/L□	12.5 ± 4.5	14.6 ± 5.4	–7.27 to 3.04	0.39	13.8 ± 5.3	14.1 ± 5.3	–5.53 to 4.92	0.90
γGTP（U/L□	19.7 ± 5.9	32.3 ± 19.5	–25.21 to -0.07	*0.05	21.3 ± 5.4	38.0 ± 23.3	–36.32 to 2.87	0.08
HDL□ mg/dl□	77.0 ± 18.0	59.9 ± 17.4	–2.67 to 36.82	0.08	66.5 ± 18.9	63.8 ± 20.2	–16.82 to 22.23	0.77
LDL□ mg/dl□	113.5 ± 17.5	119.8 ± 28.9	–29.19 to 16.50	0.56	116.5 ± 21.6	119.6 ± 31.6	–31.36 to 25.21	0.82
Hba1c (%)	5.6 ± 0.4	5.9 ± 0.5	–0.72 to 0.12	0.15	5.7 ± 0.3	6.0 ± 0.6	–0.81 to 0.22	0.20
Alb□ g/dl□	3.9 ± 0.3	3.8 ± 0.2	–0.25 to 0.42	0.57	3.9 ± 0.3	3.8 ± 0.3	-0.18 to 0.34	0.53
Glu□ mg/dl□	96.7 ± 11.5	100.4 ± 17.6	–18.22 to 10.78	0.59	101.6 ± 18.5	95.9 ± 11.2	–8.70 to 20.22	0.41
TG□ mg/dl□	83.7 ± 53.9	134.4 ± 81.9	–118.38 to 16.94	0.13	118.9 ± 73.6	117.6 ± 85.8	–79.46 to 82.03	0.97
WBC□ 10^9/L□	4.4 ± 1.0	5.5 ± 1.6	–2.37 to 0.21	0.09	4.7 ± 1.2	5.7 ± 1.8	–2.61 to 0.62	0.20
RBC□ 10^{12}/L)	4.0 ± 0.2	4.2 ± 0.3	–0.43 to 0.15	0.31	4.2 ± 0.3	4.2 ± 0.4	–0.37 to 0.31	0.83
Hb□ g/dl□	12.7 ± 0.7	12.8 ± 0.8	–0.90 to 0.67	0.75	12.8 ± 0.7	12.7 ± 0.9	–0.68 to 0.99	0.70
HCT□ %□	39.3 ± 2.4	37.9 ± 2.9	–1.29 to 4.17	0.27	38.6 ± 2.6	38.0 ± 3.1	–2.27 to 3.48	0.66
Plt□ 10^9/L□	238.2 ± 24.9	206 ± 53.9	–5.99 to 70.32	0.09	217.5 ± 38.1	214.4 ± 62.9	–52.19 to 58.34	0.90

In addition to clinical features derived from sarcopenia diagnosis, 19 participants were statistically analyzed by SMI. Comparison between the sarcopenia and non-sarcopenia groups detected significant decreases in SMI (5.0 ± 1.0 vs 6.6 ± 1.2 kg/m^2, $p < 0.05$), Body Mass Index (BMI) (20.0 ± 2.3 U/L vs 23.3 ± 3.6 kg/m^2, $p < 0.05$), serum creatinine (0.61 ± 0.22 vs 0.92 ± 0.28 mg/dl, $p < 0.05$), serum creatine kinase (CK) (81.7 ± 24.6 vs 124.4 ± 49.7 U/L, $p < 0.05$) and serum γGTP (19.7 ± 5.9 vs 32.3 ± 19.5 U/L, p < 0.05) in sarcopenia. A comparison between decreased and normal SMI groups detected significant decreases in SMI (5.2 ± 0.9 vs 7.4 ± 0.8 kg/m^2, p < 0.01, BMI (20.7 ± 2.2 vs 24.4 ± 4.0 kg/m^2, p < 0.05), serum creatinine (0.68 ± 0.23 vs 1.03 ± 0.25 mg/dl, $p < 0.01$) and serum BUN (15.6 ± 3.8 vs 22.4 ± 6.6 mg/dl, $p < 0.05$) and a significant increase in systolic blood pressure (143.9 ± 13.7 vs 129.3 ± 14.2 mmHG, $p < 0.05$). Asterisks indicate significant differences ($^*p < 0.05$). Asterisks indicate significant differences ($^{**}p < 0.01$). Abbreviation: SMI: skeletal muscle mass index; MoCA-J: Japanese version of Montreal Cognitive Assessment; TUG: Timed up and go test; BMI: Body mass index; BP: Blood pressure; CRE: creatinine; BUN, Blood urea nitrogen; CK: creatine kinase; HCT: hematocrit; CI: Confidence Interval.

Supplementary Table 2. List of 22 metabolites involved in sarcopenia.

Parameters	Peak area (10^6 AU)		*T* test	
	Sarcopenia ($N = 6$)	Non-sarcopenia ($N = 13$)	95% CI of the dif.	*p*-value
**Acetyl-carnitine**	337.73 ± 76.94	433.23 ± 99.79	–186.23 to – 4.77	*0.040
**Dimethyl-proline**	186.23± 91.05	313.38 ± 102.26	–230.09 to –24.20	*0.020
**Phenylalanine**	225.19 ± 55.70	287.35 ± 40.46	–121.27 to –3.07	*0.042
Dimethyl-arginine	38.92± 4.67	50.22 ± 13.03	–19.94 to –2.66	*0.013
N1-Methyl-histidine	21.75 ± 8.25	37.39 ± 12.08	–25.82 to –5.44	**0.005
Isovaleryl-carnitine	14.24 ± 3.85	20.57 ± 7.7	–11.95 to –0.71	*0.033
Aspartate (↑)	22.91 ± 5.65	13.64 ± 7.84	2.44 to 16.09	*0.011
myo-Inositol	13.95 ± 1.58	20.30 ± 7.43	–10.98 to –1.72	*0.011
Creatinine	15.60 ± 4.82	21.48 ± 6.64	–11.70 to –0.08	*0.047
Pantothenate	10.14 ± 3.09	17.20 ± 8.03	–12.47 to –1.65	*0.013
Hypoxanthine	4.11 ± 1.36	7.58± 4.95	–6.62 to –0.31	*0.033
Dimethyl-guanosine	2.50 ± 0.74	3.95 ± 0.81	–2.29 to –0.62	**0.003
N1-Methyl-adenosine	2.36 ±1.07	3.64 ± 1.08	–2.47 to –0.10	*0.036
2-Oxoglutarate	2.01 ± 0.49	3.01 ±1.1	–1.76 to –0.23	*0.018
Pentose-phosphate	2.13 ±0.24	2.71 ± 0.75	–1.07 to –0.09	*0.022
Succinate	1.33 ± 0.18	1.74 ± 0.45	–0.71 to –0.10	*0.012
N-Acetyl-glutamate	0.42 ± 0.14	0.58 ± 0.12	–0.31 to –0.01	*0.037
Quinolinic acid	0.26 ± 0.14	0.50 ± 0.24	–0.43 to –0.06	*0.013
4-Guanidinobutanoate	0.16 ± 0.06	0.55 ± 0.60	–0.76 to –0.03	*0.035
N1-Methyl-guanosine	0.21 ±0.09	0.32 ± 0.10	–0.21 to –0.01	*0.038
Trimethyl-tyrosine	0.05 ± 0.04	0.17 ± 0.17	–0.23 to –0.02	*0.027
cis-Aconitate	0.05 ± 0.02	0.12 ± 0.05	–0.10 to –0.04	**0.000

Metabolite peak area. High > 10^8 AU. Medium 10^7–10^8 AU. Low < 10^7 AU. 21 sarcopenia markers (acetyl-carnitine, dimethyl-proline, phenylalanine, dimethyl-arginine, N1-methyl-histidine, isovaleryl-carnitine, myo-inositol, creatinine, pantothenate, hypoxanthine, dimethyl-guanosine, N1-methyl-adenosine, 2-oxoglutarate, pentose-phosphate, succinate, N-acetyl-glutamate, quinolinic acid, 4-guanidinobutanoate, N1-methyl-guanosine, trimethyl-tyrosine, and cis-aconitate) decreased significantly. However, aspartate increased significantly in sarcopenia. Abbreviations: SMI: skeletal muscle index; CI: Confidence Interval. (↑) indicates upregulated metabolite in sarcopenia. Asterisks indicate significant differences ($^*p < 0.05$). Asterisks indicate significant differences ($^{**}p < 0.01$).

Supplementary Table 3. List of ten metabolites involved in muscle mass.

	Peak area (10^6 AU)		*T* test	
	Decreased SMI (*N* = 11)	Normal SMI (*N* = 8)	95% CI of the dif.	*p*-value
**Urate**	95.24 ± 21.23	114.54 ± 1.64	–38.18 to –0.41	*0.046
Butyro-betaine	88.02 ± 19.93	108.91± 19.46	–40.32 to –1.47	*0.037
Dimethyl-arginine	40.23 ±11.17	55.48 ± 7.04	–24.09 to –6.40	**0.002
N1-Methyl-histidine	24.73 ± 9.48	43.07 ± 9.55	–27.77 to –8.93	**0.001
Isovaleryl-carnitine	14.47 ±3.12	24.21 ± 7.76	–16.34 to –3.14	**0.009
Creatinine	16.89 ± 6.31	23.38 ± 5.27	–12.12 to –0.86	*0.026
Hippurate	12.92 ± 8.00	26.70 ± 13.19	–25.37 to –2.19	*0.020
Dimethyl-guanosine	2.98 ± 0.92	4.20 ± 0.74	–2.03 to –0.41	**0.005
2-Oxoglutarate	2.15 ± 0.53	3.45 ± 1.14	–2.27 to –0.32	*0.014
cis-Aconitate	0.07 ± 0.04	0.14 ± 0.05	–0.11 to –0.27	**0.004

Metabolite peak area. High > 10^8 AU, Medium 10^7–10^8 AU, Low < 10^7 AU. 10 metabolites (urate, butyro-betaine, dimethyl-arginine, N1-methyl-histidine, isovaleryl-carnitine, creatinine, hippurate, dimethyl-guanosine, 2-oxoglutarate, cis-aconitate) decreased significantly in sarcopenia. Abbreviations: SMI: skeletal muscle index; CI: Confidence Interval. Asterisks indicate significant differences ($^{*}p < 0.05$). Asterisks indicate significant differences ($^{**}p < 0.01$).

Supplementary Table 4. Correlation analysis between 25 sarcopenia-related markers and SMI or EFS.

Parameters	SMI			EFS		
	R	95% CI	*p* value	R	95% CI	*p* value
Isovaleryl-carnitine	0.70	0.37 to 0.88	**0.001	–0.55	–0.80 to -0.12	*0.015
**Urate**	0.58	0.18 to 0.82	**0.008	–0.54	0.10 to 0.79	*0.02
Hippurate	0.60	0.21 to 0.83	**0.006	–0.46	–0.76 to -0.01	*0.05
**Phenylalanine**	0.40	–0.07 to 0.72	0.09	–0.45	–0.75 to 0.00	0.05
Butyro-betaine	0.5	0.06 to 0.78	*0.029	–0.44	–0.75 to 0.01	0.05
**Acetyl-carnitine**	0.55	0.12 to 0.80	*0.02	–0.43	–0.74 to 0.03	0.06
N1-Methyl-guanosine	0.65	0.27 to 0.85	**0.003	–0.39	–0.71 to 0.08	0.10
Pantothenate	0.62	0.23 to 0.84	**0.005	–0.36	–0.70 to 0.11	0.13
N1-Methyl-histidine	0.58	0.17 to 0.82	**0.009	–0.3	–0.66 to 0.18	0.22
Hypoxanthine	0.52	0.09 to 0.79	*0.02	–0.29	–0.65 to 0.18	0.22
Dimethyl-guanosine	0.62	0.23 to 0.84	**0.005	–0.28	–0.65 to 0.20	0.25
Dimethyl-arginine	0.55	0.12 to 0.80	*0.02	–0.27	–0.64 to 0.20	0.26
Creatinine	0.69	0.35 to 0.87	**0.001	–0.25	–0.63 to 0.23	0.31
**Dimethyl-proline**	0.24	–0.24 to 0.63	0.32	–0.25	–0.63 to 0.23	0.31
N-Acetyl-glutamate	0.41	–0.05 to 0.73	0.08	–0.21	–0.60 to 0.26	0.38
Quinolinic acid	0.36	–0.11 to 0.70	0.13	–0.19	–0.59 to 0.28	0.44
2-Oxoglutarate	0.65	0.27 to 0.85	**0.003	–0.17	–0.58 to 0.31	0.49
Trimethyl-tyrosine	0.28	–0.20 to 0.65	0.24	–0.17	–0.58 to 0.30	0.47
Aspartate $^{(\dagger)}$	-0.45	–0.75 to –0.001	0.05	–0.17	–0.30 to 0.58	0.48
cis-Aconitate	0.57	0.16 to 0.82	*0.01	–0.16	–0.57 to 0.32	0.52
Succinate	0.59	0.19 to 0.82	**0.008	–0.12	–0.54 to 0.36	0.63
Pentose-phosphate	0.31	–0.17 to 0.67	0.20	–0.11	–0.53 to 0.36	0.65
4-Guanidinobutanoate	0.20	–0.27 to 0.60	0.41	–0.1	–0.53 to 0.37	0.68
N1-Methyl-adenosine	0.37	–0.10 to 0.71	0.12	–0.07	–0.52 to 0.40	0.76
myo-Inositol	0.52	0.09 to 0.79	*0.02	–0.04	–0.48 to 0.42	0.88

Metabolite peak area. High > 10^8 AU. Medium 10^7–10^8 AU. Low < 10^7 AU. Metabolites are ordered according to its correlation coefficient to EFS. Abbreviations: SMI: skeletal muscle index; Pearson: Pearson's correlation coefficient between a metabolite and SMI; CI: Confidence Interval; EFS: Edmonton frail scale. Asterisks indicate correlation ($^{*}p < 0.05$). Asterisks indicate correlation ($^{**}p < 0.01$).

Supplementary Table 5. Correlation analysis between 22 frailty-related markers and SMI or EFS.

	SMI			EFS		
	**R**	**95% CI**	***p* value**	**R**	**95% CI**	***p* value**
Isovaleryl-carnitine	0.70	0.37 to 0.88	**0.001	–0.54	–0.80 to –0.12	*0.015
Acetyl-carnosine	0.69	0.35 to 0.87	**0.001	–0.51	0.07 to 0.78	*0.03
Hippurate	0.60	0.21 to 0.83	**0.006	–0.46	0.01 to 0.76	*0.05
**Urate**	0.58	0.18 to 0.82	**0.009	–0.54	0.11 to 0.80	*0.02
1,5-anhydroglucitol	0.52	0.08 to 0.79	*0.02	–0.56	0.14 to 0.80	*0.01
**Proline**	0.49	0.05 to 0.77	*0.03	–0.54	0.11 to 0.80	*0.02
Methionine	0.46	0.01 to 0.76	*0.046	–0.56	0.14 to 0.80	*0.01
Leucine	0.46	0.01 to 0.75	*0.046	–0.46	0.01 to 0.75	*0.04
N3-methyl-histidine	0.45	–0.01 to 0.74	0.05	–0.19	–0.59 to 0.29	0.43
Trimethyl-histidine	0.44	–0.01 to 0.75	0.057	–0.39	–0.08 to 0.71	0.10
Isoleucine	0.42	–0.04 to 0.74	0.07	–0.45	0.14 to 0.80	*0.04
**Tryptophan**	0.37	–0.09 to 0.71	0.12	–0.57	0.15 to 0.82	*0.01
**Arginine**	0.36	–0.11 to 0.70	0.13	–0.32	–0.15 to 0.67	0.17
**Ergothioneine**	0.33	–0.14 to 0.69	0.16	–0.45	0.01 to 0.75	0.05
Adenine	0.23	–0.25 to 0.62	0.34	–0.45	–0.001 to 0.75	0.05
S-methyl-ergothioneine	0.22	–0.26 to 0.61	0.37	–0.51	0.07 to 0.78	*0.02
Ophthalmic acid	0.14	–0.34 to 0.56	0.56	–0.41	–0.06 to 0.73	0.08
2-ketobutyrate	0.14	–0.33 to 0.56	0.56	–0.29	–0.19 to 0.66	0.24
UDP-glucose (↑)	0.04	–0.41 to 0.49	0.86	–0.12	–0.55 to 0.34	0.60
Creatine (↑)	–0.08	–0.51 to 0.38	0.73	0.44	–0.74 to 0.02	0.06
N-acetyl-aspartate (↑)	–0.09	–0.52 to 0.38	0.71	0.16	–0.32 to 0.57	0.51
UDP-glucuronate (↑)	–0.15	–0.56 to 0.32	0.54	0.47	–0.76 to -0.02	*0.04

Metabolite peak area. High > 10^8 AU. Medium 10^7–10^8 AU. Low < 10^7 AU. Metabolites are ordered according to its correlation coefficient to SMI. Abbreviations: SMI: skeletal muscle index; Pearson: Pearson's correlation coefficient between a metabolite and SMI; CI: Confidence Interval. Asterisks indicate correlation ($^*p < 0.05$). Asterisks indicate correlation ($^{**}p < 0.01$).
